# Longevity, demographic characteristics, and socio-economic status are linked to triiodothyronine levels in the general population

**DOI:** 10.1073/pnas.2308652121

**Published:** 2024-01-04

**Authors:** Ralph I. Lawton, Bernardo L. Sabatini, Daniel R. Hochbaum

**Affiliations:** ^a^Harvard Medical School, Boston, MA 02115; ^b^Department of Neurobiology, Harvard Medical School, Boston, MA 02115; ^c^HHMI, Chevy Chase, MD 20815

**Keywords:** thyroid, T3, mortality, socioeconomic outcomes, health demographics

## Abstract

The hypothalamic–pituitary–thyroid (HPT) axis is fundamental to human biology, exerting central control over energy expenditure and body temperature. However, the consequences of normal physiologic HPT-axis variation in populations without diagnosed thyroid disease are poorly understood. Using nationally representative data from the 2007 to 2012 National Health and Nutrition Examination Survey, we explore relationships with demographic characteristics, longevity, and socio-economic factors. We find much larger variation across age in free T3 than other HPT-axis hormones. T3 and T4 have opposite relationships to mortality: free T3 is inversely related and free T4 is positively related to the likelihood of death. Free T3 and household income are negatively related, particularly at lower incomes. Finally, free T3 among older adults is associated with labor both in terms of unemployment and hours worked. Physiologic TSH/T4 explain only 1.7% of T3 variation, and neither are appreciably correlated to socio-economic outcomes. Taken together, our data suggest an unappreciated complexity of the HPT-axis signaling cascade broadly such that TSH and T4 may not be accurate surrogates of free T3. Furthermore, we find that subclinical variation in the HPT-axis effector hormone T3 is an important and overlooked factor linking socio-economic forces, human biology, and aging.

## Significance

Thyroid hormone influences many aspects of human physiology and behavior. However, the consequences of normal variation in thyroid levels in general populations are unknown. Here, we find strong relationships between triiodothyronine (T3) levels and health and socioeconomic outcomes, notably with mortality, income, and labor participation (employment status and hours worked)

The hypothalamic–pituitary–thyroid (HPT) axis exerts central control over core components of human biology, including energy expenditure and body temperature regulation. Abnormal levels of thyroid hormone (either hypo- or hyper-thyroid) result in metabolic dysregulation and myriad psychiatric disease symptoms (1–7). Despite the clinical focus on hyper- and hypo-thyroidism, most individuals’ thyroid hormones have “set-points” that vary subclinically, and laboratory reference ranges are large and therefore insensitive to normal variation in thyroid function (8–10). The prominence of both metabolic and psychological phenotypes in thyroid disease states suggests that the HPT axis could govern aspects of health and human behavior within normal, subclinical variation as well. Further, given the declines in HPT-axis function and metabolism with aging, these sub-clinical set-points may be important mediators of well-being among older populations (11–13). However, the role of normal physiological variation of the HPT-axis in human behavior, socio-economic forces, and population health remains poorly understood, particularly with respect to the HPT-axis effector hormone T3 (8).

The canonical HPT-axis signaling cascade begins with the hypothalamus stimulating the pituitary gland to release TSH (thyroid stimulating hormone), TSH stimulates the thyroid gland to secrete primarily T4 (thyroxine), and some T3 (triiodothyronine), and the exact T3/T4 ratio is dependent on TSH concentration (14, 15). The vast majority of T3 is produced in the periphery of the body, where T4 is converted into T3 by deiodinases DIO1 and DIO2. T3 is considered to be the active hormone, with ~10- to 30-fold higher affinity for thyroid receptors than the prohormone T4 (16). Changes in T3 levels alter the function of tissues throughout the body (17). Clinically, hypothyroidism can lead to lethargy, weight gain, hypothermia, and depression, whereas hyperthyroidism can lead to weight loss, hyperthermia, agitation, restlessness, and hyperactivity, sleep disruption, and other manic-like symptoms.

Emerging lines of research have investigated potential interplay between thyroid function, behavior, and the environment. New findings show that thyroid hormone may coordinate exploratory behaviors and metabolic responses to changes in environmental resource availability (18). Other work finds alterations in thyroid function with contemporaneous and early-life stress, nutrition and food insecurity, and long-run economic studies of iodine supplementation indirectly implicate thyroid function in labor force participation and income (19–23). While population-based studies of thyroid function are sparse, mounting evidence suggests that socio-economic forces have the potential to both shape or be shaped significantly by thyroid function. Yet, little attention has been given to sub-clinical variation in HPT-axis function.

Understanding the role of the HPT-axis in aging and population health is complicated by mixed evidence from studies of the HPT-axis and longevity. Although low T3 and T4 levels have been linked to adverse mortality outcomes in hospitalized or cardiac patients, the role of HPT-axis hormones in non-clinical populations is less clear (24, 25). Evidence from euthyroid patients visiting clinics in South Korea found both free T3 and free T4 to be protective against mortality, whereas other studies of euthyroid patients in Europe have found free T4 to be negatively related to mortality (26–28). Interventions with levothyroxine (synthetic T4) have not been protective against mortality in older adults but may be beneficial in younger adults (29).

Here, we use nationally representative cross-sectional data from the USA to study the relationships between HPT-axis function in the general population across the physiological range with demographic characteristics, seasonality, socio-economic forces, and mortality. We use simultaneous measures of TSH, free T4, and free T3 for each respondent enabling assessment of HPT-axis hormones at the time of measurement, and correlate their levels with key dimensions of well-being, especially as people age.

## Results

We combined data on TSH, free T4, and free T3 from 7,626 adults over age 20 the 2007 to 2012 survey waves of the National Health and Nutrition Examination Survey (NHANES). Weighted summary statistics are in *SI Appendix*, Table S1. To account for potential untreated clinical hypo- and hyper-thyroidism, we drop individuals outside the top and bottom 1% of the sample (n = 691), as well as individuals on thyroid disorder medications (n = 433). For all regression results, we estimate three models to examine relationships between covariates and thyroid hormones. We specify a base model conditioning on demographic characteristics (including age), survey wave, smoking, and medication use and extend this model to include measures of socio-economic status (SES), as well as measures of health that may be related to thyroid function (*Data and Methods*). We focused on free T3 and T4 since protein-bound T4 and T3 are not thought to be biologically active. Simultaneous measures of TSH, free T4, and free T3 in each survey participant enables localization within this chain of hormones where the relationships between the HPT-axis with population health and socio-economic forces may occur.

### Demographic Characteristics and Seasonality.

**Fig. 1.**
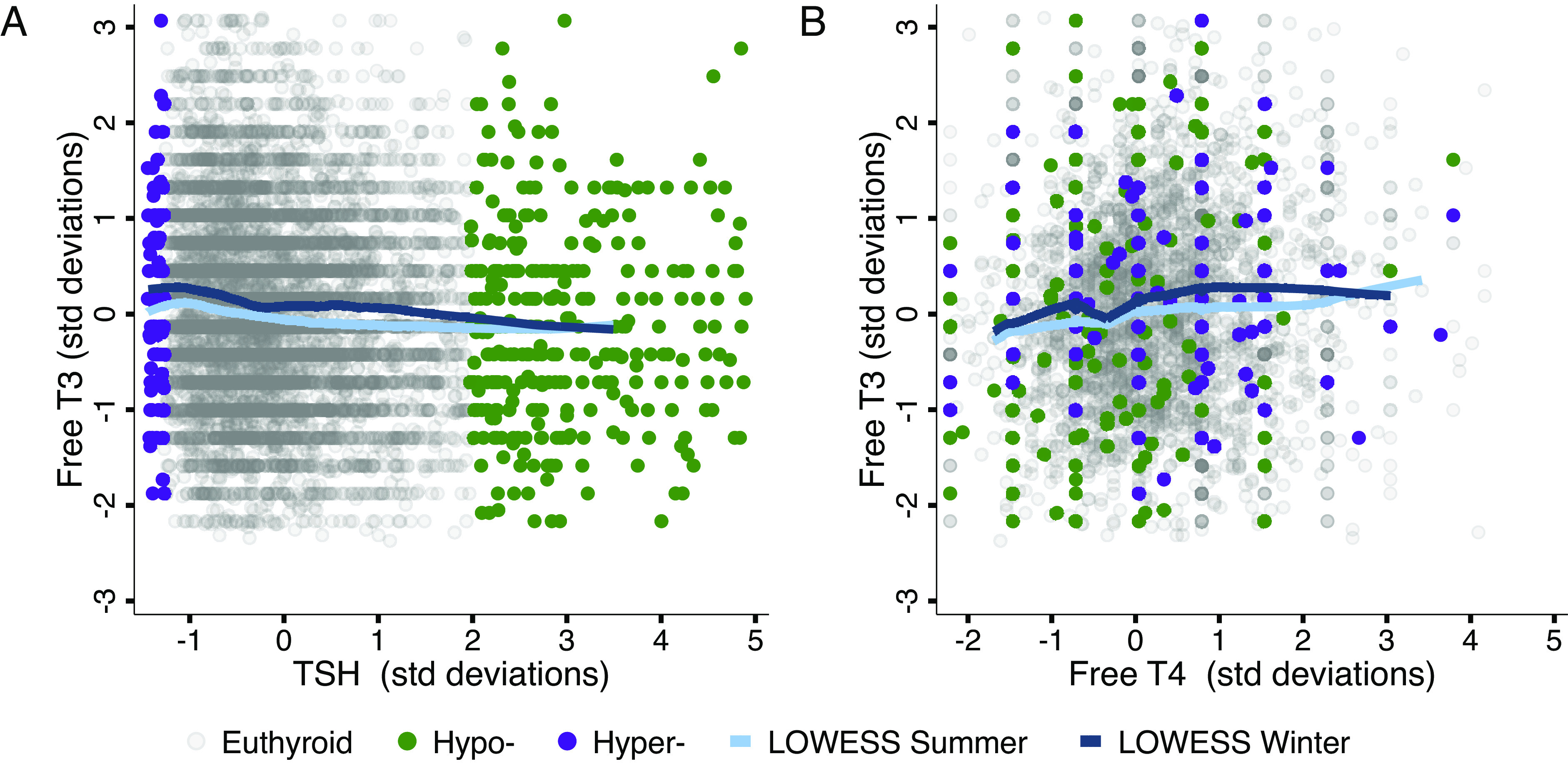
Inter-relationships between TSH, free T4, and free T3 in SDs. (*A*) Scatter plot showing TSH and free T3 for the same individual, in terms of SDs of the adult population distribution. (*B*) Scatter plot showing free T4 and free T3 for the same individual, in terms of SDs of the adult population distribution. Euthyroid, hypo-, and hyper- thyroid individuals in both panels classified by TSH (hypo: TSH > 4.1 mIU/L, hyper: TSH < 0.4 mIU/L). Nonparametric LOWESS estimates shown stratifying by summer measurement (May 1 to October 31). *SI Appendix*, Figs. S1 and S2 display parallel results in nonstandardized units, and stratifying overt and subclinical hypothyroidism.

We sought to confirm previous findings relating basic demographic characteristics and seasonality to HPT-axis function to indicate the robustness of our chosen dataset. Consistent with evidence from the 2001 NHANES that studied TSH but not free T3 or T4, we find that males and females have statistically indistinguishable TSH levels. We also find small differences in free T4 which are not robust to controls for other health measures (Table 1) (30).^*^ Additionally, we find that males have 0.5 SDs (0.17 pg/mL) higher free T3 levels (*P* < 0.001), consistent with data from clinical samples (31, 32). Examining differences by race replicates prior findings with respect to TSH (Table 1) (30). Black Americans have 0.4 SDs lower TSH and 0.1 SDs lower free T3 than non-Hispanic white Americans (*P* < 0.01). Hispanic Americans have 0.1 SD lower TSH, 0.2 SD higher free T3 (*P* < 0.01), and 0.1 SD higher free T4 (*P* < 0.05), though free T4 results are not robust to SES controls. Finally, evidence from mammalian studies implicates seasonality in thyroid function, as do recent meta-analyses of human thyroid studies, attributed largely to the role of thyroid function in thermoregulation (33–35). Consistent with these studies, we find evidence for lower free T3 during the summer (Table 1 and Fig. 1). Therefore, the 2007 to 2012 waves of the NHANES recapitulate canonical findings of HPT-axis function in a nationally representative sample.

**Table 1.** Demographic, socio-economic, and health relationships with standardized thyroid-axis hormones

	[1]	[2]	[3]	[4]	[5]	[6]	[7]	[8]	[9]	[10]	[11]	[12]
	Base			Base and SES			Base, SES, health and health behavior			Other thyroid hormones		
Model specification:	Free T3	Free T4	TSH	Free T3	Free T4	TSH	Free T3	Free T4	TSH	Free T3	Free T4	TSH
Free T3											0.16***	−0.01
											(0.02)	(0.01)
Free T4										0.15***		−0.11***
										(0.02)		(0.02)
TSH										−0.01	−0.10***	
										(0.01)	(0.01)	
(1) Male	0.51***	0.08***	0.01	0.52***	0.08***	0.01	0.57***	0.03	0.01	0.56***	−0.06	0.02
	(0.02)	(0.03)	(0.03)	(0.02)	(0.03)	(0.03)	(0.04)	(0.04)	(0.05)	(0.04)	(0.05)	(0.05)
(1) Black	−0.12***	−0.04	−0.40***	−0.17***	−0.04	−0.40***	−0.19***	−0.08	−0.39***	−0.18***	−0.08	−0.40***
	(0.04)	(0.05)	(0.03)	(0.04)	(0.05)	(0.04)	(0.04)	(0.05)	(0.04)	(0.04)	(0.05)	(0.04)
(1) Hispanic	0.19***	0.09**	−0.13***	0.12**	0.02	−0.10**	0.07	0.04	−0.11**	0.06	0.02	−0.11**
	(0.04)	(0.04)	(0.03)	(0.05)	(0.05)	(0.05)	(0.05)	(0.05)	(0.05)	(0.06)	(0.05)	(0.05)
(1) Race non-white, black, or hispanic	−0.00(0.06)	0.34***(0.05)	−0.16***(0.04)	−0.01(0.07)	0.26***(0.05)	−0.13**(0.05)	0.03(0.08)	0.23***(0.05)	−0.10(0.06)	−0.00(0.08)	0.22***(0.05)	−0.08(0.06)
(1) Summer measurement	−0.09*(0.05)	−0.03(0.06)	0.00(0.03)	−0.10**(0.05)	−0.02(0.06)	0.00(0.03)	−0.11**(0.05)	−0.02(0.06)	0.01(0.03)	−0.11**(0.05)	−0.01(0.06)	0.01(0.03)
ln(Real HH income)				−0.06***(0.02)	−0.01(0.02)	−0.03*(0.01)	−0.05***(0.02)	−0.02(0.02)	−0.00(0.01)	−0.05***(0.02)	−0.01(0.02)	−0.01(0.01)
(1) College Ed.				−0.10***(0.04)	0.07**(0.03)	0.01(0.04)	−0.05(0.04)	0.05*(0.03)	0.03(0.05)	−0.06(0.04)	0.07**(0.03)	0.03(0.05)
Constant	0.31***	−0.14*	−0.00	0.96***	−0.03	0.28*	0.90***	0.08	0.06	0.89***	−0.05	0.07
	(0.06)	(0.07)	(0.05)	(0.19)	(0.21)	(0.16)	(0.21)	(0.22)	(0.16)	(0.20)	(0.21)	(0.16)
Observations	7,626	7,626	7,626	7,626	7,626	7,626	7,021	7,021	7,021	7,021	7,021	7,021
R-squared	0.216	0.056	0.052	0.221	0.058	0.053	0.234	0.066	0.062	0.252	0.097	0.073

SEs in parentheses. Models utilize weights to account for the population sampling probabilities of the NHANES and use linearized SEs.

All models conditional on age, medication use, smoking, and survey wave. SES models conditional on household size and nativity. Health models conditional on Height, waist circumference, hours of sleep, iodine levels, grams of carbohydrates per day, grams of protein per day, grams of fat per day, and %HbA1c. T3, T4, and TSH outcomes expressed as SDs. ***P < 0.01, **P < 0.05, *P < 0.1.

### Relationships between TSH, T4, and T3.

While T4 and T3 levels are dependent on TSH concentrations, no population-representative studies exist that measure all three canonical HPT-axis hormones simultaneously within individuals to quantitatively parse their relationships. Evidence from clinical samples suggests that HPT-axis hormones are not strongly predictive of one another, as free T4 and even TSH can be unreliable measures of sub-clinical but symptomatic hypothyroidism (13, 36, 37). Further, 5 to 10% of hypothyroid individuals treated with levothyroxine (LT4) that successfully normalize TSH levels remain symptomatic, suggesting that normal TSH and T4 levels can be insufficient to restore normal T3-mediated physiology (38, 39).

We therefore directly compared TSH, free T4, and free T3 within individuals to evaluate the correlation between these HPT-axis molecules. We find that the relationships between TSH (Fig. 1*A* and *SI Appendix*, Fig. S1*A*) and free T4 (Fig. 1*B* and *SI Appendix*, Fig. S1*B*) with free T3 are weak, explaining 0.6% and 1.3% of the variation in free T3 respectively, and 1.7% combined (*SI Appendix*, Table S2).^†^ While we exclude individuals who are being actively treated for thyroid conditions, some individuals in the general population still meet TSH and free T4-based threshold-based criteria for hyper-, hypo-, and subclinical hypo-thyroidism.^‡^ Even with these clinical thresholds, TSH and free T4 poorly stratify free T3 levels (*SI Appendix*, Fig. S2). Therefore, free T3 levels cannot be reliably predicted by TSH and free T4 levels, and direct measurement of free T3 is likely vital to properly stratify the effects of HPT-axis variation on clinical and non-clinical populations.

### Aging and Longevity.

**Fig. 2.**
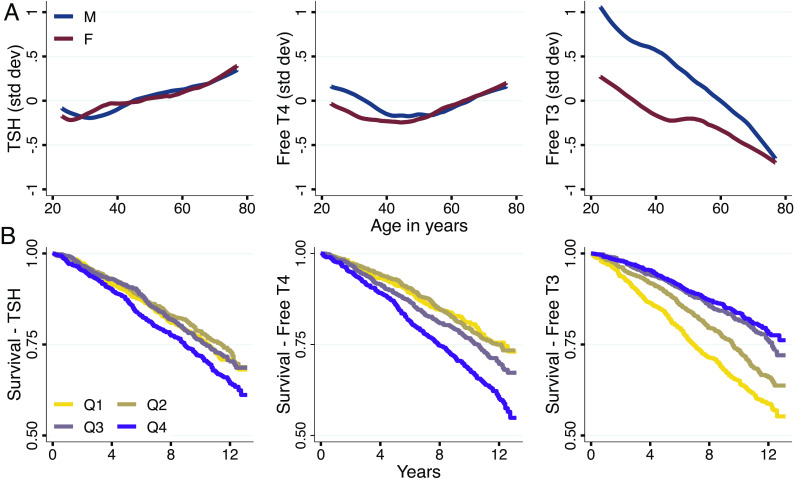
Thyroid hormones, age, and longevity. (*A*) Weighted nonparametric LOWESS estimates of the relationships between thyroid hormones and age among adults, stratified by sex. (*B*) Weighted survival curves since measurement date for each quartile of TSH, free T4, and free T3, among adults over age 50 (Q1: 0 to 25%, Q2: 25 to 50%, Q3: 50 to 75%, Q4: 75 to 100% within each hormone’s distribution).^§^

Age is one of the strongest predictors of clinical hypothyroidism, and age-related HPT-axis function has been implicated in the decline of metabolism and increase in adiposity as adults age (30, 40, 41). However, prior work has only focused on TSH and free T4 levels, which are commonly used in the clinical diagnosis of thyroid disease. Non-parametric models stratified by sex show the relationships between all three standardized HPT-axis hormones and age (Fig. 2*A*). Free T3 exhibits substantially more variation across age than either free T4 or TSH, with over twice the variation between oldest and youngest than TSH. Free T3 declines with age, consistent with its role as the active effector molecule of the HPT-axis, whose function declines with age. While total T3 follows similar patterns to free T3, the relationships with age are attenuated (*SI Appendix*, Fig. S3), likely due to unaccounted variability of T3 binding proteins. TSH levels steadily increase over time, whereas the free T4 pattern is nonlinear, declining until approximately age 50 and then increasing. Given that hypothyroidism is typically diagnosed clinically as high TSH levels and low T4, the simultaneous increases later in life in both TSH and free T4 could potentially confound diagnosis of clinical and sub-clinical hypothyroidism (6).

Given the prominent variation with age, we next examined whether HPT-axis hormones are predictive of longevity. Using mortality data through the end of 2019 linked to the NHANES, we use Cox proportional-hazards models to evaluate the relationships between HPT-axis hormones and longevity in a non-clinical population. Kaplan–Meier curves separated by quartile of each hormone are shown in Fig. 2*B*. We focused on adults over 50 (n = 3,603), among whom there were 981 deaths in this time period. TSH is not linked to mortality, but increasing levels of free T3 and decreasing levels of free T4 are protective from mortality (hazard ratios 0.88 and 1.26, respectively, *P* < 0.01) and retain their coefficients conditional on socioeconomic status (SES) and important markers of health including measures of adiposity like waist circumference (*SI Appendix*, Table S3). Higher T3 quartiles have slightly higher BMI than lower ones (Q4: 29.50, Q1: 28.62), but as BMI is linked to higher mortality, these should bias mortality estimates the opposite direction of the T3 relationship. Total T3 has a similar relationship to mortality as free T3, but total T4 is attenuated significantly from free T4’s mortality relationship (*SI Appendix*, Fig. S3 and Table S4). As illness can affect thyroid hormone levels and directly affect mortality, we evaluate the potential role of illness by extending our model to include covariates for flu, colds, or GI illness in the past 30 d, as well as measures of inflammation using C-Reactive Protein from the first two waves of data. Mortality results are robust to these controls, though some statistical power is lost when only using the first two waves (*SI Appendix*, Table S5).

Notably, these biomarkers are not predictive of longevity simply in the short term but continue to stratify mortality through the long-term endpoint (Fig. 2*B*). These results parallel the age-related changes in T3 and T4 levels and suggest that they may be important factors related to longevity. Further, the opposing relationships of T3 and T4 levels with mortality (high T3 and low T4 are protective from mortality), mechanistically implicate changes in the relationships between T3 and T4 levels with age, potentially due to deiodinase-mediated conversion of T4 to T3 peripherally or altered T4/T3 production by the thyroid gland.

### SES and Employment.

**Fig. 3.**
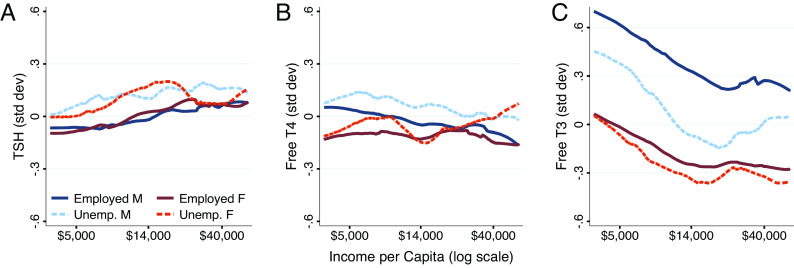
Thyroid hormones, household income, and unemployment. Panels *A*, *B*, and *C* show relationships for TSH, free T4, and free T3, respectively. Panels *A*, *B*, and *C* show relationships for TSH, free T4, and free T3, respectively. Weighted nonparametric LOWESS estimates of the relationship between thyroid hormones and the natural logarithm of real (2007 dollars) household income per capita are displayed (within the central 5th to 95th percentiles of the distribution), stratified by sex and employment status at the time of measurement. Data from 2007 to 2012 NHANES.

We examined the correlation between household resources and thyroid function. Conditional on prior discussed covariates, we find a robust negative relationship between household income and free T3, but no relationship between household income and free T4 (Table 1 and Fig. 3). Although there is a negative relationship between income and TSH (Fig. 3*A*), the estimate is not robust to controlling for other health factors that may affect the HPT-axis, nor to an extended model that controls for free T3; however, the free T3 relationship is robust to controlling TSH (Table 1). The negative relationship between free T3 and SES persists with respect to alternative measures of SES including household income per capita, and income-to-poverty ratios that account for household size, state, and year-specific poverty thresholds (*SI Appendix*, Table S6). Similar models for total T3 find similar, but less precisely estimated relationships between income and total T3 than free T3 (*SI Appendix*, Fig. S7 and Table S8).

The negative relationship between free T3 and household resources is stronger at lower income levels (Fig. 3*C*). We estimate the location of a “kink” in the relationship between income and free T3 (Fig. 3*C*) and conduct statistical tests for the significance of a threshold effect relative to a linear model (*SI Appendix*, *Supplement B1*) (42). Our analysis identifies the threshold at $22,735 per capita and finds the kink model to be a significantly better fit than the linear model (*P* < 0.001). In addition, $22,735 is approximately half of average income per capita in 2007, suggesting an interaction between stressors associated with lower-income households and T3 (21).

Although this observed correlation cannot be interpreted causally, the relationship is nearly identical for employed and unemployed adults across both genders (Fig. 3*C*), suggesting that potential links between T3 and earnings from employment are not driving the income–T3 correlation directly but rather implicating a household-level factor related to available resources. The relationship is meaningfully large in magnitude, similar to or larger than for other biomarkers with important income-gradients like cholesterol, % HbA1C, blood pressure, and C-reactive protein (*SI Appendix*, Table S7) (43, 44).

Although household resources and free T3 are inversely related, Fig. 3 shows free T3, but not free T4 or TSH, is lower among unemployed men. We used ordinary least squares (OLS) and logit models to explore potential relationships between free T3 and employment status among men. Since age is a key moderator of both employment status and free T3, we specify a fully-interacted model of our full specification’s covariates interacted with age, that can account for co-variation of free T3 and employment status as individuals age (*Data and Methods*). We find evidence that at older ages, free T3 is a key moderator of employment status (Table 2; see *SI Appendix*, Table S9 for similar models using total T3). Logit models find that for each decade increase in age, a 1 SD increase in T3 is linked to a 17% increase in the odds of employment. Nonparametric explorations bear this effect out: Among adults under 40, there is no apparent relationship between free T3 and employment status; however, among adults over 40, there is a clear positive relationship, which is particularly salient at lower levels of free T3 (*SI Appendix*, Fig. S4). It is possible that the varied relationships as people age may be related to retirement decision making as people approach older ages.

**Table 2.** Relationships between free T3 and labor market outcomes

	[1]	[2]	[3]	[4]	[5]	[6]	[7]	[8]	[9]	[10]	[11]
	Employed								Hours worked		
	Base model		Base model		Add SES		Add health		All men	Employed men	Employed in high-activity job
	OLS	Logit	OLS	Logit	OLS	Logit	OLS	Logit	OLS	OLS	OLS
Free T3	0.00	1.02	0.02	1.04	0.01	1.05	0.00	1.02	0.08	0.55	1.88***
	(0.01)	(0.06)	(0.01)	(0.07)	(0.01)	(0.07)	(0.01)	(0.07)	(0.56)	(0.58)	(0.69)
Age (10 y)	−0.11***	0.56***	−0.13***	0.46***	−0.22**	0.44	−0.19*	0.46	−3.96	1.73	−4.27
	(0.01)	(0.03)	(0.01)	(0.04)	(0.09)	(0.29)	(0.10)	(0.34)	(5.25)	(4.96)	(7.37)
T3 * Age			0.03***	1.17***	0.02***	1.11***	0.01**	1.07*	0.97***	1.09***	1.03***
			(0.00)	(0.04)	(0.01)	(0.04)	(0.01)	(0.04)	(0.26)	(0.30)	(0.31)

Models utilize weights to account for the population sampling probabilities of the NHANES and use linearized SEs. All models conditional on age, medication use, smoking, and survey wave. SES models conditional on household size and nativity. Health models conditional on Height, waist circumference, hours of sleep, iodine levels, grams of carbohydrates per day, grams of protein per day, grams of fat per day, and %HbA1c. Coefficients come from a fully interacted model with age and the described covariates. SEs in parentheses. T3, T4, and TSH outcomes expressed as SDs. ***P < 0.01, **P < 0.05, *P < 0.1.

This association with free T3 is apparent with respect to both employment status and hours worked. Regression models estimating the association between free T3 and the number of hours worked overall and conditional on employment show increases not only in labor market participation but also in the numbers of hours worked (Table 2 and *SI Appendix*, Figs. S5 and S6). These relationships with hours worked are strongest among those employed in “high-activity” jobs, classified as manual or service jobs, among whom there is a strong association with free T3 and the number of hours worked irrespective of age (45). The association between employment and free T3 is consistent with the effects of increased free T3 on energy expenditure—effects that are observed clinically in the context of hyperthyroidism with increased metabolism in addition to manic and hyperactive psychiatric symptoms.

Notably, across all models, we can explain a much larger (~2–5x) proportion of variation in free T3 than in free T4 or TSH (Table 1). While we can explain 24% of the variation in free T3, we only can explain 6 to 7% of the variation in free T4 or TSH with the same models.

## Discussion

In a non-clinical sample representative of the US population, we find that free T3 is much more strongly related to all domains studied—age, sex, seasonality, household income, employment, and longevity—than the other molecules of the HPT-axis. The relative strength of these relationships reinforces free T3’s privileged role as the primary effector of the HPT-axis. Further, these results suggest that for population research, and likely for clinical surveillance, increased focus on free T3 as a primary readout of HPT-axis function is warranted.

These findings have particular salience for the well-being of aging populations. Although it is known that HPT-axis function declines with age, measured variation in free T3 is much larger than the variation in free T4 or TSH, and only T3 steadily declines with age. Furthermore, free T4 and free T3 diverge at older ages, when free T4 increases and free T3 continually decreases. One intriguing possibility is that this may reflect age-related decline in the efficiency of T4 to T3 conversion by deiodinases (12, 46–48). Alternatively, changes in the ratio of T4/T3 production from the thyroid gland may play a role (49). Given known declines in HPT-axis function as people age, and the typical use of free T4 and TSH in diagnosis of hypo-thyroidism, diverging patterns in free T4 and free T3 with age have significant implications for the proper diagnosis and HPT-axis assessment at older ages: Older adults with symptoms of hypothyroidism may have high free T4 levels despite low free T3.

The divergence between free T3 and free T4 at older ages is paralleled in our mortality findings. While increasing free T3 is protective against mortality, increasing free T4 is linked to higher levels of mortality, suggesting that the age-related decline in T3 and T3/T4 ratio have important impacts on longevity. These results parallel similar findings on free T3, T4, and deiodinase function in hospitalized and frail patients but suggest further functional importance at sub-clinical levels in the general population (50). Poor deiodinase function, or altered expression, at older ages may also reconcile why levothyroxine (synthetic L-T4) therapy does not improve mortality outcomes among the oldest patients with sub-clinical hypothyroidism but may be helpful at younger ages (29). Recent findings have suggested a potentially important clinical role for free T3 measurement, as well as benefits of combination liothyronine and levothyroxine therapy in highly symptomatic patients, which is consistent with an important role of age-related changes in T3 and the T3/T4 ratio (14, 51).

We find a robust negative relationship between household resources and free T3, relating SES to thyroid hormone profiles in the general population. We do not see this pattern for either free T4 or TSH. This evidence is consistent across employed and unemployed household members of both sexes, suggesting that direct effects of income generation are not driving the observed pattern. Given the potential for unmeasured confounders, and the nature of cross-sectional data used, it is difficult to definitively assign a mechanism. Nonetheless, the relationship is primarily present at lower levels of income, suggesting a threshold at which the association of income with free T3 levels diminish potentially consistent with a response to economic stress (21). While T3 levels are known to be sensitive to nutrition (which varies with SES), we find the income-free T3 pattern is robust to controls for carbohydrate and protein intake (Table 1), though there may be other unobserved components of diet that are important.

Stratifying our income relationships by employment status does not reveal differential relationships. Instead, doing so emphasizes a significant drop in free T3 levels among unemployed versus employed males, suggesting separate relationships between free T3 with income and employment, respectively (Tables 1 and 2 and Fig. 3). This may have important implications for well-being, especially among older males. We find that at ages 40 and older, free T3 is significantly linked to the likelihood of being employed. This relationship shows similar patterns in employment status as well as hours worked among those who are employed. However, we are not able to observe all the members of a household, and while our results are robust to controls for household income from a potential working partner, we are unable to take the employment decision-making of other household members fully into account. Our findings suggest that variation in levels of T3 among the general population influences individuals’ willingness to engage and be active, particularly at older ages. These relationships are consistent with T3’s role in governing energy expenditure, the prominence of hyperactive psychiatric symptoms in hyperthyroidism, and recent evidence from animal models on the role of free T3 on exploratory activity and behavior. Further, it aligns with economic evidence suggesting treating overt clinical hypothyroidism as well as evidence that iodine-fortification leads to improved employment outcomes, particularly later in life (18, 23, 52).

These results underscore the potential importance of measuring and understanding free T3 as the primary effector molecule of the HPT-axis. It is important to note that the more typically measured biomarkers for thyroid function (T4 and TSH) are poorly linked to free T3 levels. We document weak relationships between TSH, T4, and T3 and find less than 2% of the variation in free T3 is explainable with these other measures. Improved methods for measurement and further investigation of the role of free T3 in clinical conditions may be high-yield.

The evidence presented here on the central role of free T3 also gives insight into the potential mechanisms involved. Free T3 is much more strongly related than other HPT measures to canonical HPT-axis variation like seasonality, sex, and age, as well as SES and employment. Furthermore, T3 diverges from free T4 with respect to age and longevity. The results suggest potential mechanisms related to the ratio between T3/T4. Among them, the efficiency of conversion between T4 and T3 by deiodinases, or direct changes in thyroid secretion. Further studies of the relationship between deiodinase activity and social forces and aging could elucidate critical biology, while new therapeutics targeting T3/T4 ratios may be more beneficial for a significant cohort of older individuals currently treated with levothyroxine.

## Data and Methods

### Data.

We combine data from the 2007 to 2008, 2009 to 2010, and 2011 to 2012 waves of the NHANES, a sample representative of the United States population when weighted (https://wwwn.cdc.gov/nchs/nhanes/Default.aspx). These are the only recent waves of the NHANES study that measure free T3, free T4, and TSH.^¶^ While all adults in the 2007 to 2008 NHANES were eligible for HPT-axis measures, only sub-samples of the 2009 to 2010 and 2011 to 2012 waves were selected to have HPT-axis measures collected (53). We focus on adults over age 20 at the time of survey and exclude women who were pregnant at the time of assessment. We also exclude adults who are on medications for thyroid disorders, which can distort the relationships between TSH, T4, and T3. To account for potential untreated clinical hyper and hypothyroidism, we drop individuals outside the top and bottom 1% of the sample.^#^

We focus on the key hormones of the HPT-axis: TSH, free T4, and free T3. Canonically, the hypothalamus stimulates the pituitary gland to release TSH, TSH stimulates the thyroid gland to secrete primarily T4 and some T3, and the vast majority of T3 is produced in the periphery of the body, where T4 (considered a prohormone with limited activity) is converted into the active hormone T3 by deiodinase enzymes (5). T3 and T4 both circulate in free (active) and protein-bound forms (inactive). Across all NHANES waves used, thyroid hormone concentrations were assessed using a Beckman Coulter Access 2 immunoassay, validation documentation publicly available from the National Center for Health Statistics (NCHS) (53).

Linked mortality information through 2019 is sourced from the NCHS public-use linked mortality dataset. Since mortality among individuals under 50 is very rare, we focus our mortality analysis on adults over age 50 at the time of measurement.

Age is reported by the respondent. In these data, it is top-coded at age 80. Race and ethnicity data, education data, household size, smoking behavior, and country of origin is based on self-report. “Summer” measurement is defined as measurement in the 6-mo period between May 1 and October 31. Employment status is based upon whether respondents reported they were “working at a job or business” in the last week. Income is assessed at the household level, is imputed at $115,000 for top-coded values over $100,000 and is adjusted across waves for inflation to 2007 dollars.

Where used, clinical cutoffs for hypothyroidism, sub-clinical hypothyroidism, and hyperthyroidism were defined as follows: hypothyroidism (TSH > 4.1 mIU/L, free T4 < 15th percentile), sub-clinical hypothyroidism (TSH > 4.1 mIU/L, free T4 > 15th percentile), and Hyperthyoidism was defined as (TSH < 0.4 mIU/L). Classifications of high and low TSH approximate 2012 guidelines from the AACE/ATA, and typical laboratory reference ranges from recent empirical work (6, 54, 55). Percentiles were used for free T4 to conservatively categorize hypothyroidism from sub-clinical hypothyroidism given the lack of clear guidelines for sub-clinical hypothyroidism and free T4, as older work like the Colorado Thyroid Disease Prevalence Study or NHANES III used total T4 levels, and very few adults (<0.5%) had free T4 levels over 1.8 ng/dL as suggested by Kim et al. (6, 54, 56, 57).

### Statistical Analysis.

To account for the complex sampling design of the NHANES, all models were adjusted for sampling weights, clustering, and stratification. Sample weights take into account the response rates in each wave, which were 75.4%, 77.3%, and 69.5% in 2007 to 2008, 2009 to 2010, and 2011 to 2012, respectively. Since the thyroid hormone data in the 2009 to 2010 and 2011 to 2012 waves were 1/3 subsamples with specific subsample weights to represent the full population in those waves, the weights were divided by three, such that within each wave the weights still are population-representative, but that across waves individual observations are similarly weighted. Analyses were conducted using the SVY routine with linearized SEs in Stata/SE 17.0.

Sampling in the NHANES, and the questionnaires used, are described at length by the NCHS and are publicly available (53). Briefly, the sampling frame for the NHANES represents the total noninstitutionalized civilian population. Primary sampling units were first selected at random from US counties, and census blocks (or combinations of smaller blocks) within those areas were then selected at random. Within these areas, households and then individuals were selected at random with some oversampling for target populations. The probabilities of being selected are accounted for in the sampling weights such that the final sample, when weighted, is representative of the US population. While response rates are high in these waves, differential attrition on unobserved characteristics not accounted for by survey weights, particularly as related to health status or thyroid function, could potentially bias results. Nonetheless, our results are robust to alternative sampling weights, and alternative winsorization of individuals with extreme levels of thyroid markers.

In order to examine associations between the HPT-axis hormones and age, a known driver of thyroid function, we used LOWESS to explore nonparametric relationships (58).

Next, we used a series of OLS models to 1) establish core demographic relationships between age, gender, race, and seasonality of measurement and 2) examine the potential relationships between HPT-axis hormones and socio-economic gradients, focusing on household resources. We specify three models, all of which are conditional on thyroid-related medication use, statin use, and wave. For the first model, we examine relationships between age, sex (self-reported sex assigned at birth), race, and seasonality on HPT-axis hormones, conditional on medication use, wave, and smoking behavior. Due to non-linearities in HPT-axis hormones and age, particularly free T4, we model age semi-parametrically with a series of dummy indicators for each decade.^||^ Second, we extend the first model to real household income, college education, household size, and nativity. Finally, we extend the model further to investigate whether our demographic and economic relationships of interest are confounded by common factors that affect the HPT-axis, namely body composition, sleep, iodine levels, nutrition, and HbA1c as a proxy for long-term blood glucose.

In order to study mortality, we apply the same series of models described above. We use Cox proportional hazards models specified on the months that have passed between an interview date and mortality in order to account for varying time between each wave and the 2019 mortality data endpoint.

We also investigate the interplay between the HPT-axis and employment status. Specifying both OLS and logistic regression models conditional on the same covariates described above, we investigate the relationships between HPT-axis hormones and the likelihood an individual is currently employed. Since both employment status and HPT-axis hormones have strong relationships with age, we specify fully interacted models that allow the relationship of HPT-axis hormones with employment to vary by age.

In order to supplement our regression analyses of the relationships between HPT-axis hormones with income and employment, we use LOWESS to examine nonlinearities in these relationships (58). With respect to employment, in order to visualize the interaction between age and free T3 with employment, we stratify into three age categories, standardize free T3 within each group, and display the LOWESS models of employment probability. Observing that the nonparametric relationship between income and free-T3 is downward-sloping at lower income levels and functionally flat at higher levels, we follow Hansen (42) to estimate the location of a potential threshold at which the slopes change and test for whether the regression fit on either side of the threshold are significantly different from the base linear model (*SI Appendix*, *Supplement B1*).

## Supplementary Material

Appendix 01 (PDF)

## Data Availability

Previously published data were used for this work (https://www.cdc.gov/nchs/nhanes/index.htm) (53).

## Footnotes

^*^While Aoki et al. did not study free T4, our results on free T4 are similar to theirs on total T4. Conditional on age and race, Aoki et al. finds small but statistically significant differences in total T4 by sex. We find similar results in free T4 with our base model, similar to Aoki et al., but conditioning on further health measures renders the estimate nonsignificant.

^†^In particular, medications for thyroid disorders can distort relationships between TSH, T4, and T3—alternative specifications that do not drop individuals on such medications have weaker relationships between the three hormones—only 1% of the variation in free T3 can be explained.

^‡^Hyper-thyroid criteria includes 1.47% of the sample, hypo-thyroid criteria are met by 1.09%, and subclinical hypo-thyroid criteria are met by 3.28% of the sample. As discussed further below, our core findings are robust to dropping individuals who meet these criteria (*SI Appendix*, Tables S10–S13).

^§^TSH: Q1 = 0.201 to 1.156 uIU/mL, Q2 = 1.156 to 1.73 uIU/mL, Q3 = 1.731 to 2.544 uIU/mL, Q4: 1 = 2.545 to 7.405 uIU/mL. Free T4: Q1 = 0.5 to 0.69 ng/dL, Q2 = 0.7 to 0.79 ng/dL, Q3 = 0.8 to 0.89 ng/dL, Q4 = 0.9 to 1.35 ng/dL. Free T3: Q1 = 2.33 to 2.8 pg/mL, Q2 = 2.81 to 3 pg/mL, Q3 = 3.01 to 3.25 pg/mL, Q4 = 3.26 to 4.2 pg/mL.

^¶^While we are not fully certain why TSH, and to a lesser extent T4, have been the focus of prior population-based literature, the CDC stopped collecting data on T4 and T3 after 2012, saying in email correspondence that there was no further public-health support for T3 collection.

^#^Our results are not sensitive alternative specifications that winsorize at 5 or 10%, nor are they sensitive to dropping all individuals with clinical hypo and hyperthyroidism (*SI Appendix*, Tables S10–S13). Weighted summary statistics are presented in *SI Appendix*, Table S1.

^||^Nonetheless, results are robust to linear instead of semi-parametric controls for age (*SI Appendix*, Table S14).
