# Supplementary material for: Longevity, demographic characteristics, and socio-economic status are linked to triiodothyronine levels in the general population
Source: Proc Natl Acad Sci U S A. 2024 Jan 4;121(2):e2308652121. doi: 10.1073/pnas.2308652121 (PMC10786306; doi:10.1073/pnas.2308652121)
Supplement: Supplementary file 1 — Appendix 01 (PDF) [file pnas.2308652121.sapp.pdf]

PNAS

Supporting Information for

Longevity, demographic characteristics, and socio-economic status are linked to triiodothyronine levels in the general population.

Ralph I. Lawton1, Bernardo L. Sabatini1,2,3*, & Daniel R. Hochbaum1,2 *

1 Harvard Medical School, Boston, MA, USA
2 Department of Neurobiology, Harvard Medical School, Boston, MA, USA
3 Howard Hughes Medical Institute, Chevy Chase, MD, USA

Correspondence:
daniel_hochbaum@hms.harvard.edu, bernardo_sabatini@hms.harvard.edu

This PDF file includes:

Figures S1 to S7
Supporting figures and text B1
Tables S1 to S14

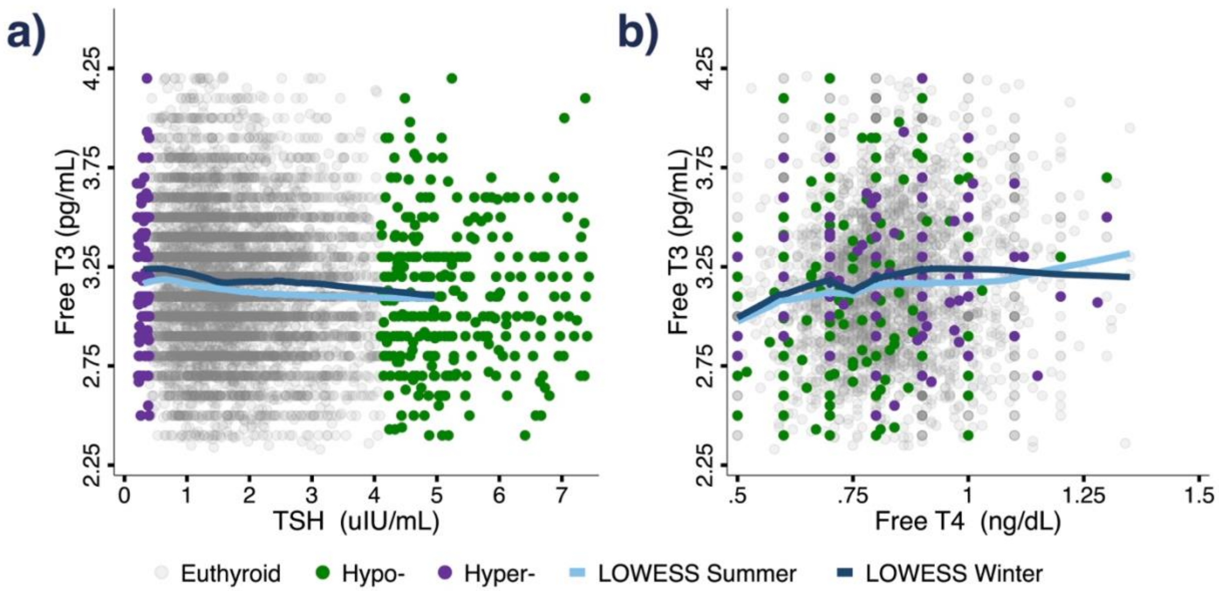

Figure S1. Inter-relationships between TSH, free T4, and free T3 in clinical units. a) Scatter plot showing TSH and free T3 for the same individual. b) Scatter plot showing free T4 and free T3 for the same individual. Euthyroid, hypo-, and hyper- thyroid individuals in both panels classified by TSH (hypo: TSH>4.1 mIU/L, hyper: TSH<0.4 mIU/L). Non-parametric LOWESS estimates shown stratifying by summer measurement (May 1-Oct. 31).

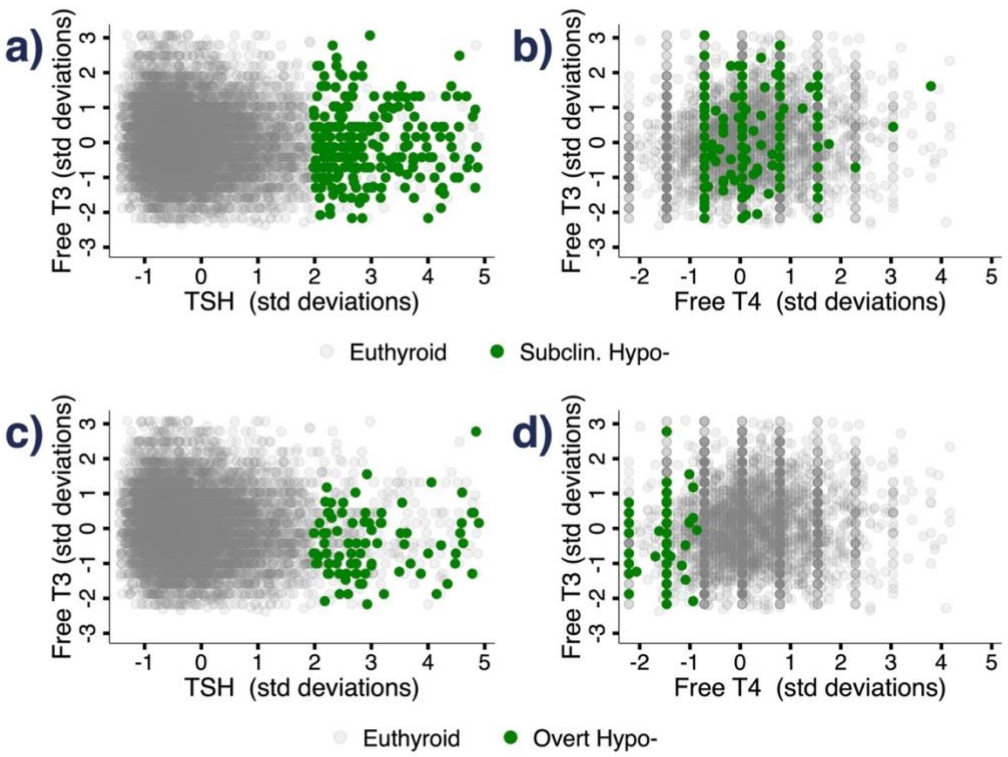

Figure S2. Inter-relationships between TSH, free T4, and free T3 in standard deviations, stratifying overt and subclinical hypothyroidism. a) Scatter plot showing TSH and free T3 for the same individual, in terms of standard deviations of the adult population distribution, highlighting subclinical hypothyroidism in green (TSH>4.1mIU/L, free T4>0.7ng/dL). b) Scatter plot showing free T4 and free T3 for the same individual, in terms of standard deviations of the adult population distribution, highlighting subclinical hypothyroidism in green. c) Scatter plot showing TSH and free T3 for the same individual, in terms of standard deviations of the adult population distribution, highlighting overt hypothyroidism in green (TSH>4.1mIU/L, free T4<0.7ng/dL). d) Scatter plot showing free T4 and free T3 for the same individual, in terms of standard deviations of the adult population distribution, highlighting overt hypothyroidism in green. In a regression framework, (Supplement table S2), adults that would be classified as hypothyroid (high TSH low free T4), are only 1/3 of a standard deviation lower in free T3 levels conditional on our base model. Adults that would be classified with sub-clinical hypothyroidism (high TSH, "normal" free T4), stratify free T3 even more poorly. Even hyperthyroid classifications (low TSH), performs poorly, with functionally no relationship between TSH and free T4 to free T3.

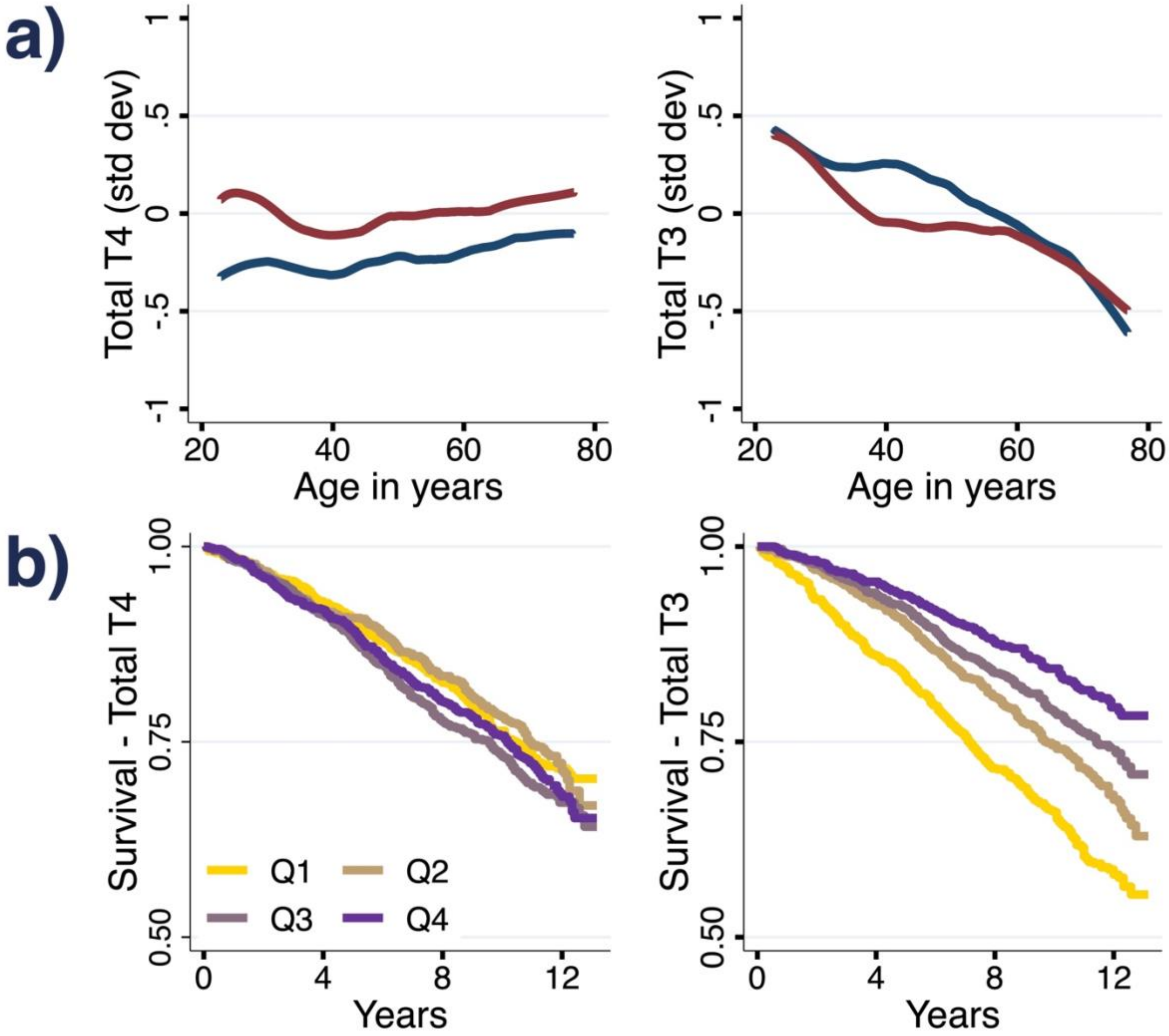

Figure S3. Total T4, Total T3, age, and mortality. **A)** weighted non-parametric LOWESS estimates of the relationships between thyroid hormones and age among adults, stratified by sex. **B)** weighted survival curves since measurement date for each quartile of total T4 and total T3, among adults over age 50 (Q1: 0-25%, Q2: 25-50%, Q3: 50-75%, Q4: 75-100% within each hormone's distribution).

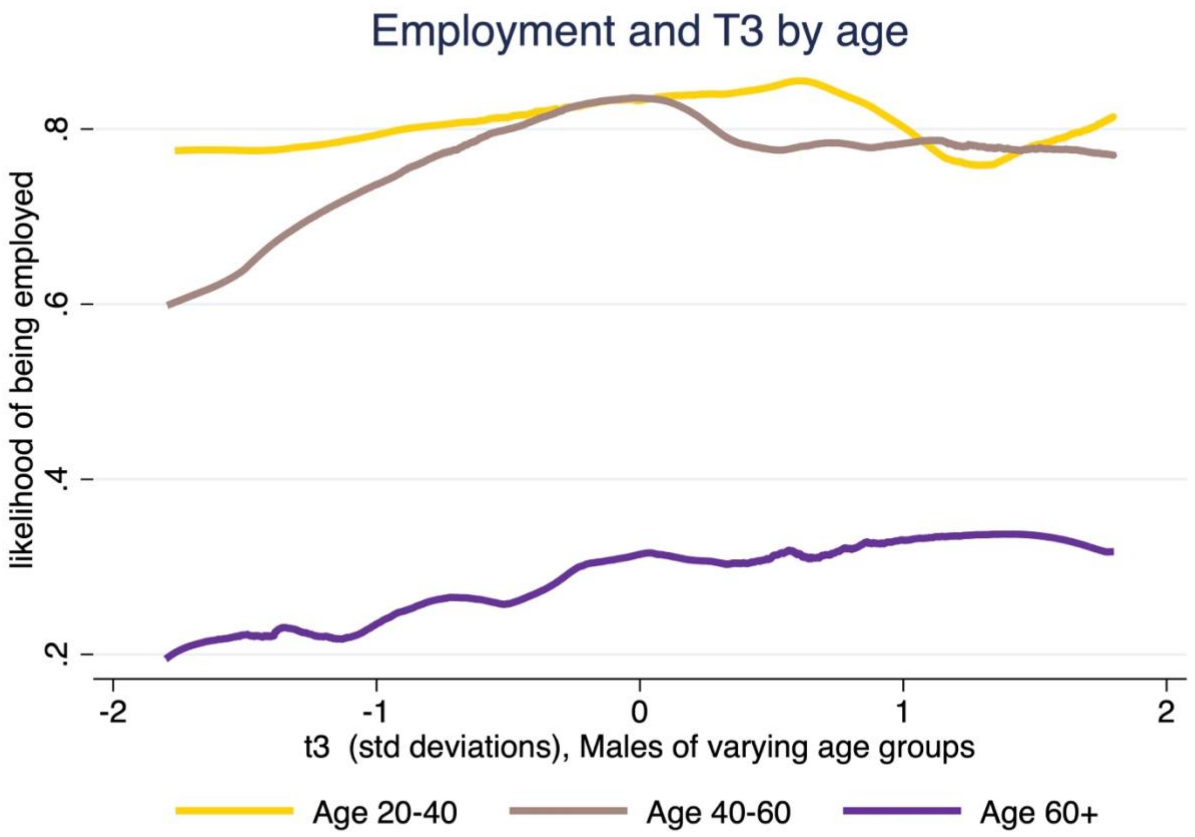

Figure S4. Employment likelihood and free T3 among males of varying age groups. Weighted non-parametric LOWESS estimates of the relationship between free T3 and employment status at the time of measurement are shown. T3 standard deviations are calculated within each age group set. Data from 2007-2012 NHANES.

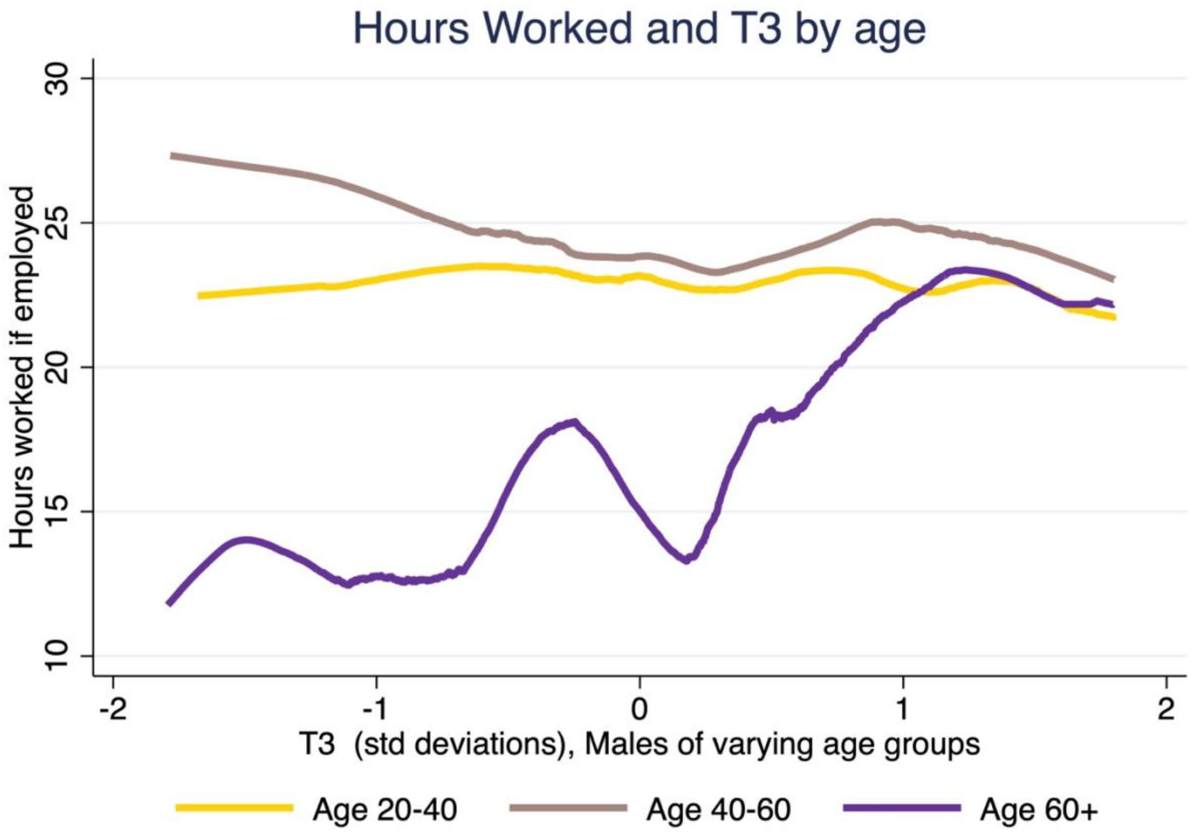

Figure S5. Hours worked and free T3 among males of varying age groups. Weighted non-parametric LOWESS estimates of the relationship between free T3 and hours worked among employed adults are shown. T3 standard deviations are calculated within each age group set. Data from 2007-2012 NHANES.

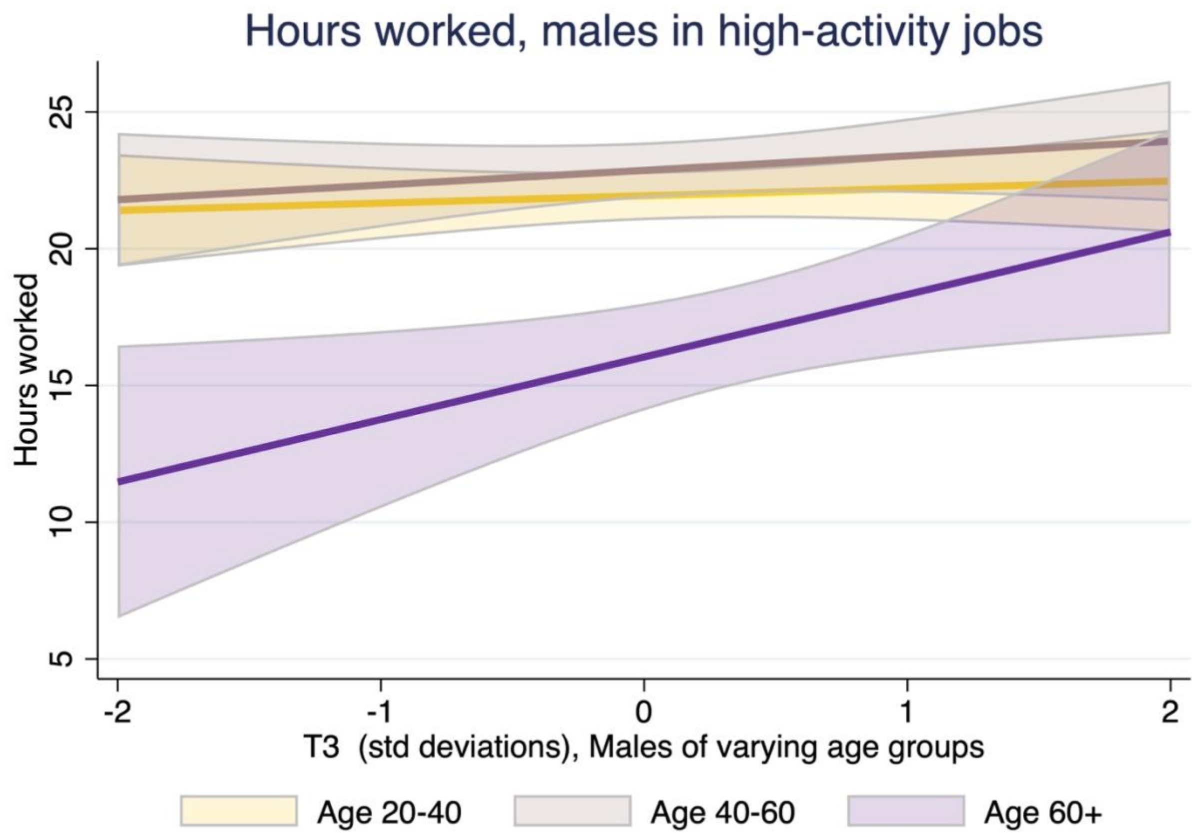

Figure S6. Hours worked and free T3 among males of varying age groups working in 'high activity' manual or service industry jobs. Weighted linear regression estimates of the relationship between free T3 and hours worked among employed adults are shown. 90% confidence intervals shown. Linear regression used instead of LOWESS due to restricted sample size. T3 standard deviations are calculated within each age group set. Data from 2007-2012 NHANES.

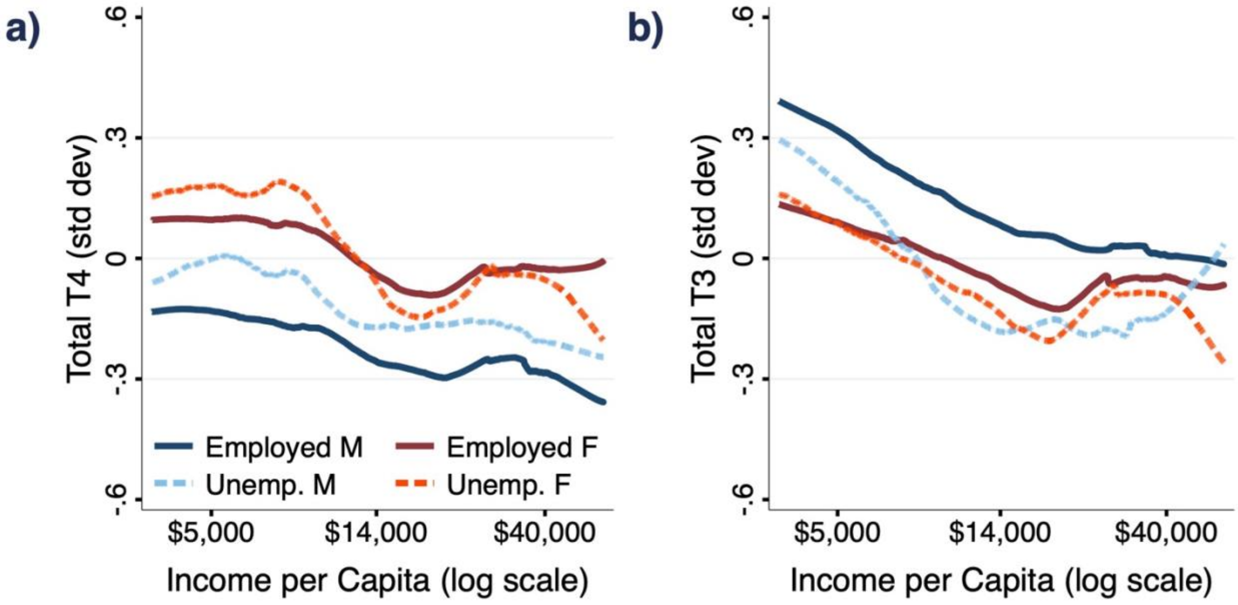

Figure S7. Total T4, Total T3, household income, and unemployment. Weighted non-parametric LOWESS estimates of the relationship between Total T4 and T3 and the natural logarithm of real (2007 dollars) household income per capita are displayed, stratified by sex and employment status at the time of measurement. Data from 2007-2012 NHANES.

Supplement B1. Regression-Kink Tests

Analysis for a regression kink were conducted following Hansen (2017). In the first-stage, visualized below, we minimize the least-squares criterion after iteratively estimating threshold regressions as specified below, where z_i represents the free T3 outcome, y_i represents the natural log of real income per capita, τ is the location of the income threshold (which we are trying to estimate), $I(.)$ is an indicator function, β_1 and β_2 are slope parameters, and ε_i represents the error term.

$$(1) \qquad z_i = \beta_0 + \beta_1 y_i + \beta_2 (y_i - \tau) \times I(y_i - \tau > 0) + \varepsilon_i$$

We iteratively estimate (1) in increments of 0.01 with respect to the natural log of real income per capita, and plot the MSE in the figure below, identifying the point at which the least-squared error is minimized, in this case at $22,735.

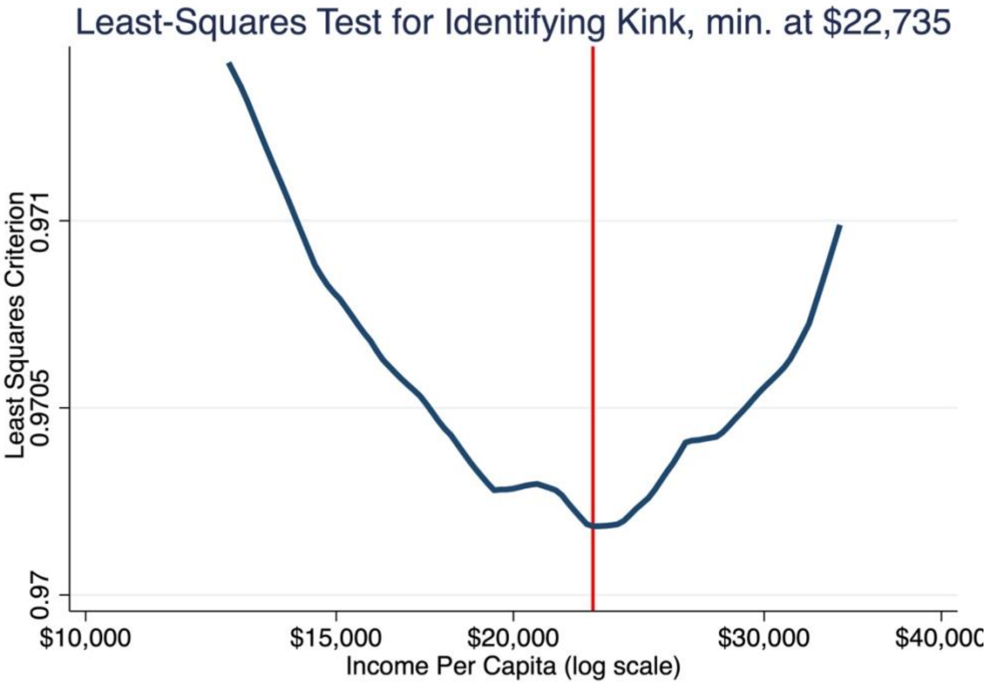

Having identified the optimal location for a kink, we first calculate an F-like statistic as described in Hansen (2017). In order to conduct inference on this statistic, we use a boot-strap approach with 1,000 replications that calculates simulated F-like statistics, and compare these to our calculated F-like statistic for our optimal threshold regression. In doing so, we find strong evidence rejecting the null hypothesis that $\beta_2 = 0$, with $p<0.001$.

Table S1: Summary statistics from NHANES analytic sample, Age 20+

	Unadjusted NHANES Weights								Analytical NHANES Weights							
	Males				Females				Males				Females			
	Mean	SD	Max	Min	Mean	SD	Max	Min	Mean	SD	Max	Min	Mean	SD	Max	Min
TSH (uIU/mL)	1.830	(1.060)	7.430	0.200	1.812	(1.080)	7.380	0.201	1.857	(1.101)	7.430	0.200	1.861	(1.119)	7.380	0.201
Free T4 (ng/dL)	0.798	(0.127)	1.350	0.500	0.791	(0.123)	1.350	0.500	0.790	(0.125)	1.350	0.500	0.780	(0.123)	1.350	0.500
Free T3 (pg/mL)	3.258	(0.339)	4.210	2.360	3.081	(0.310)	4.200	2.330	3.265	(0.336)	4.210	2.360	3.080	(0.312)	4.200	2.330
Age in years	46.57	(15.88)	80	21	47.33	(16.23)	80	21	46.52	(15.78)	80	21	47.15	(16.25)	80	21
ln(real household income)	10.74	(0.754)	11.65	7.743	10.62	(0.823)	11.65	7.743	10.75	(0.754)	11.65	7.743	10.63	(0.819)	11.65	7.743
Proportion Employed	0.673	(0.469)	-	-	0.558	(0.497)	-	-	0.688	(0.463)	-	-	0.559	(0.497)	-	-
Proportion Black	0.0955	(0.294)	-	-	0.122	(0.327)	-	-	0.0944	(0.292)	-	-	0.120	(0.324)	-	-
Proportion Hispanic	0.148	(0.355)	-	-	0.140	(0.347)	-	-	0.145	(0.352)	-	-	0.136	(0.343)	-	-
Proportion measured in summer	0.586	(0.492)	-	-	0.595	(0.491)	-	-	0.599	(0.490)	-	-	0.613	(0.487)	-	-
Height (m)	1.759	(0.0767)	2.045	1.421	1.622	(0.0707)	1.942	1.345	1.759	(0.0765)	2.045	1.421	1.623	(0.0706)	1.942	1.345
Waist Circumference (m)	1.013	(0.149)	1.782	0.654	0.953	(0.157)	1.703	0.591	1.012	(0.149)	1.782	0.654	0.950	(0.157)	1.703	0.591
Proportion wave 2009-2010	0.337	(0.473)	-	-	0.328	(0.469)	-	-	0.204	(0.403)	-	-	0.199	(0.399)	-	-
Proportion wave 2011-2012	0.336	(0.472)	-	-	0.348	(0.476)	-	-	0.203	(0.402)	-	-	0.211	(0.408)	-	-
Proportion high school completed	0.255	(0.436)	-	-	0.254	(0.435)	-	-	0.263	(0.440)	-	-	0.260	(0.439)	-	-
Proportion some college	0.296	(0.456)	-	-	0.277	(0.447)	-	-	0.280	(0.449)	-	-	0.281	(0.449)	-	-
Proportion college completed	0.275	(0.447)	-	-	0.278	(0.448)	-	-	0.276	(0.447)	-	-	0.263	(0.440)	-	-
Sample Size	3,985				3,641				3,985				3,641			

Models utilize sampling weights to account for the population sampling probabilities of the NHANES.
Adjusted weights reflect division by 3 of weights in later waves, such that the mean individual in each wave is weighted approximately the same.
This adjustment accounts for the 1/3 sub-sampling of the individuals with thyroid measures in the later waves.

Table S2. Relationships Between HPT-axis Hormones and Clinical Cutoffs

	[1]	[2]	[3]	[4]	[5]	[6]	[7]	[8]
	Free T3							
	All Adults						All Adults	
	No Controls			Base Model			No Controls	Base Model
Free T4	0.12***		0.11***	0.13***		0.13***		
	(0.01)		(0.01)	(0.01)		(0.01)		
TSH		-0.08***	-0.06***		-0.02*	-0.01		
		(0.01)	(0.01)		(0.01)	(0.01)		
Hypothyroid							-0.51***	-0.31***
							(0.12)	(0.10)
Subclinical Hypothyroid							-0.08	0.01
							(0.09)	(0.08)
Hyperthyroid							-0.03	-0.01
							(0.10)	(0.10)
R-squared	0.013	0.006	0.017	0.231	0.216	0.231	0.003	0.217

Models utilize weights to account for the population sampling probabilities of the NHANES, and use linearized standard errors.

Adults not on meds excludes all adults on thyroid-related medications.

All models conditional on age, medication use, smoking, and survey wave, and base model covariates from table 1.

Clinical category definitions: Hypothyroid (TSH>4.1 & Free T4<15th %ile), Subclinical Hypothyroid (TSH>4.1 & T4>15th %ile), Hyperthyroid (TSH<0.4)

T3, T4, and TSH outcomes expressed as standard deviations

*** p<0.01, ** p<0.05, * p<0.1

Table S3. Relationships between Thyroid Hormones and Mortality, Age 50+

	[1]	[2]	[3]	[4]	[5]	[6]
	Base Model				Add SES	Add Health
	Mortality					
Free T3	0.88**			0.86***	0.85***	0.89*
	(0.04)			(0.04)	(0.04)	(0.05)
Free T4		1.23***		1.26***	1.27***	1.26***
		(0.05)		(0.06)	(0.05)	(0.05)
TSH			1.01	1.05	1.05	1.05
			(0.03)	(0.03)	(0.03)	(0.04)

Models utilize weights to account for the population sampling probabilities of the NHANES, and use linearized standard errors.
Hazard Ratios come from a Cox proportional hazards model conditional on the described covariates. Standard errors in parentheses.
All models conditional on age, medication use, smoking, and survey wave, and covariates from base model in table 1. SES models conditional on household size and nativity. Health models conditional on Height, waist circumference, hours of sleep, iodine levels, grams of carbohydrates per day, grams of protein per day, grams of fat per day, and %HbA1c.
Sample restricted to over 50 years old to focus on mortality. Mortality data through 2019.
T3, T4, and TSH outcomes expressed as standard deviations
*** $p<0.01$, ** $p<0.05$, * $p<0.1$

Table S4. Relationships between Total T3, Total T4, and Mortality, Age 50+

	[1]	[2]	[5]	[6]
	Base Model		Add SES + Health	
	Mortality			
Total T3	0.86***		0.91*	
	(0.04)		(0.04)	
Total T4		1.15***		1.12**
		(0.05)		(0.05)

Models utilize weights to account for the population sampling probabilities of the NHANES, and use linearized standard errors. Hazard Ratios come from a Cox proportional hazards model conditional on the described covariates. Standard errors in parentheses.

Sample restricted to over 50 years old to focus on mortality. Mortality data through 2019.

Total T3 and T4 expressed as standard deviations

*** p<0.01, ** p<0.05, * p<0.1

Table S5. Relationships between Thyroid Hormones, Mortality, and Illness, Age 50+

	[1]	[2]
	Base Model with Illness Covariates	
	w/o CRP	with CRP
	Mortality	
Free T3	0.86***	0.88**
	(0.04)	(0.04)
Free T4	1.25***	1.19***
	(0.06)	(0.05)
TSH	1.05	1.00
	(0.03)	(0.04)
Observations	3,603	2,959

Models utilize weights to account for the population sampling probabilities of the NHANES, and use linearized standard errors.
Hazard Ratios come from a Cox proportional hazards model conditional on the described covariates. Standard errors in parentheses.
C-Reactive Protein only in first two waves, reducing sample size
Illness covariates include whether or not respondent has had a cold, gastrointestinal illness, or flu within the past 30 days.
Sample restricted to over 50 years old to focus on mortality. Mortality data through 2019.
T3, T4, and TSH outcomes expressed as standard deviations
*** $p<0.01$, ** $p<0.05$, * $p<0.1$

Table S6. Alternate Income Specifications for Demographic, Socio-economic, and Health Relationships with Standardized Thyroid-Axis Hormones

	[1]	[2]	[3]	[4]	[5]	[6]
Model Specification:	Per-Capita Income	Income-Poverty Ratio	Per-Capita Income	Income-Poverty Ratio	Per-Capita Income	Income-Poverty Ratio
	Free T3	Free T3	Free T4	Free T4	TSH	TSH
ln(Real Per Capita HH Income)	-0.04**		-0.02		-0.01	
	(0.02)		(0.02)		(0.01)	
Poverty Index Ratio		-0.02**		-0.01		-0.00
		(0.01)		(0.01)		(0.01)
Observations	7,021	6,429	7,021	6,429	7,021	6,429
R-squared	0.234	0.236	0.066	0.066	0.062	0.061

Standard errors in parentheses. Models utilize weights to account for the population sampling probabilities of the NHANES, and use linearized standard errors.

Poverty Index Ratio is calculated using HHS guidelines, dividing household income by household size, state, and year specific poverty thresholds

Full model presented models conditional on covariates from full model described in table 1

Table S7. SES Regressions of Other Biomarkers (Standardized)

Model Specification:	[1]	[2]	[3]	[4]	[5]	[6]
	Base & SES					
	HDL	Non-HDL	%HbA1C	Sys. BP	Dias. BP	CRP
(1) Male	-0.40***	0.11***	0.03***	0.26***	0.26***	-0.17***
	(0.02)	(0.02)	(0.01)	(0.02)	(0.02)	(0.03)
(1) Black	0.15***	-0.18***	0.14***	0.18***	0.06	0.19***
	(0.02)	(0.03)	(0.02)	(0.04)	(0.04)	(0.05)
(1) Hispanic	-0.06**	0.02	0.10***	0.03	-0.07	0.10*
	(0.03)	(0.03)	(0.01)	(0.03)	(0.05)	(0.05)
(1) Race non-white, Black, or Hispanic	-0.07*	-0.05	0.07***	-0.01	0.01	-0.13***
	(0.03)	(0.05)	(0.02)	(0.04)	(0.06)	(0.04)
(1) Summer measurement	-0.04*	0.00	0.01	0.02	0.03	-0.02
	(0.02)	(0.02)	(0.01)	(0.02)	(0.03)	(0.03)
ln(Real HH Income)	0.05***	-0.01	-0.04***	-0.02	0.03	-0.07***
	(0.01)	(0.02)	(0.01)	(0.02)	(0.02)	(0.03)
(1) College Ed.	0.14***	-0.12***	-0.06***	-0.16***	-0.07**	-0.11***
	(0.02)	(0.02)	(0.01)	(0.03)	(0.03)	(0.03)
Constant	-0.73***	-0.50***	-0.36***	-0.28	-0.61**	0.71**
	(0.13)	(0.16)	(0.07)	(0.18)	(0.25)	(0.26)
Observations	7,625	7,625	7,613	7,100	7,100	6,195
R-squared	0.149	0.119	0.164	0.223	0.125	0.033

Standard errors in parentheses. Models utilize weights to account for the population sampling probabilities of the NHANES, and use linearized standard errors.

All models conditional on age, medication use, smoking, household size, nativity, and wave.

All biomarkers standardized so outcomes represent changes in number of standard deviations of a given biomarker

CRP not present in 2011-2012 survey wave, lowering sample size

Table S8. Demographic, Socio-economic, and Health Relationships with Standardized Total T3

Model Specification:	[1]	[2]	[3]	[4]	[5]	[6]
	Total T3			Total T4		
	Base	SES	Full	Base	SES	Full
(1) Male	0.11***	0.11***	0.16***	-0.24***	-0.24***	-0.22***
	(0.02)	(0.02)	(0.04)	(0.02)	(0.02)	(0.04)
(1) Black	-0.11**	-0.15***	-0.16***	0.10*	0.07	0.01
	(0.05)	(0.05)	(0.05)	(0.05)	(0.05)	(0.05)
(1) Hispanic	0.16***	0.09**	0.04	0.25***	0.16***	0.10*
	(0.04)	(0.04)	(0.04)	(0.04)	(0.05)	(0.05)
(1) Race non-white, Black, or Hispanic	-0.14**	-0.15*	-0.13	0.21***	0.15***	0.13**
	(0.07)	(0.08)	(0.08)	(0.04)	(0.05)	(0.05)
(1) Summer measurement	-0.03	-0.04	-0.06	-0.17***	-0.18***	-0.19***
	(0.05)	(0.05)	(0.06)	(0.05)	(0.05)	(0.05)
ln(Real HH Income)		-0.04**	-0.04*		-0.04**	-0.02
		(0.02)	(0.02)		(0.02)	(0.02)
(1) College Ed.		-0.14***	-0.10**		-0.04	-0.01
		(0.04)	(0.04)		(0.03)	(0.03)
Constant	0.25***	0.77***	0.75***	0.01	0.41**	0.39**
	(0.07)	(0.24)	(0.24)	(0.05)	(0.19)	(0.19)
Observations	7,626	7,626	7,021	7,599	7,599	6,995
R-squared	0.078	0.083	0.100	0.046	0.048	0.071

Standard errors in parentheses. Models utilize weights to account for the population sampling probabilities of the NHANES, and use linearized standard errors.

All models conditional on age, medication use, smoking, and survey wave. SES models conditional on household size and nativity. Health models conditional on Height, waist circumference, hours of sleep, iodine levels, grams of carbohydrates per day, grams of protein per day, grams of fat per day, and %HbA1c.

Table S9. Relationships between Total T3 and Labor Market Outcomes

	[1]	[2]	[3]	[4]	[5]	[6]	[7]	[8]	[9]	[10]	[11]
	Employed								Hours Worked		
	Base Model		Base Model		Add SES		Add Health		All Men	Employe d Men	Employe d in High-Activity Job
	OLS	Logit	OLS	Logit	OLS	Logit	OLS	Logit	OLS	OLS	OLS
Free T3	0.01	1.02	0.01	1.02	0.01	1.04	0.00	1.00	0.04	0.35	0.58
	(0.01)	(0.07)	(0.01)	(0.06)	(0.01)	(0.06)	(0.01)	(0.06)	(0.56)	(0.51)	(0.77)
T3 * Age			0.02***	1.13***	0.01**	1.08*	0.01	1.05	0.46	0.52	0.12
			(0.01)	(0.04)	(0.01)	(0.05)	(0.01)	(0.05)	(0.33)	(0.34)	(0.61)
Age (10 years)			-0.12***	0.48***	0.01**	1.08*	-0.18*	0.49	-3.62	2.26	-3.02
			(0.01)	(0.04)	(0.01)	(0.05)	(0.10)	(0.35)	(5.38)	(4.87)	(7.20)

Models utilize weights to account for the population sampling probabilities of the NHANES, and use linearized standard errors.
Coefficients come from a fully interacted model with age and the described covariates. Standard errors in parentheses.
T3, T4, and TSH outcomes expressed as standard deviations
*** p<0.01, ** p<0.05, * p<0.1

Table S10. Robustness of Demographic, Socio-economic, and Health Relationships to Winsorization

	[1]	[2]	[3]	[4]	[5]	[6]	[7]	[8]	[9]	[10]	[11]	[12]	[13]	[14]	[15]	[16]	[17]	[18]
Model Specification:	Free T3						Free T4						TSH					
	Base - 6%	Base - 10%	SES - 6%	SES - 10%	Health - 6%	Health - 10%	Base - 6%	Base - 10%	SES - 6%	SES - 10%	Health - 6%	Health - 10%	Base - 6%	Base - 10%	SES - 6%	SES - 10%	Health - 6%	Health - 10%
(1) Male	0.44***	0.42***	0.45***	0.43***	0.48***	0.46***	0.08***	0.08***	0.07***	0.07***	0.05	0.04	-0.00	-0.00	0.00	0.00	0.01	0.01
	(0.02)	(0.02)	(0.02)	(0.02)	(0.04)	(0.04)	(0.02)	(0.02)	(0.02)	(0.02)	(0.04)	(0.04)	(0.02)	(0.02)	(0.02)	(0.02)	(0.04)	(0.04)
(1) Black	-0.08***	-0.08***	-0.12***	-0.12***	-0.13***	-0.13***	-0.03	-0.02	-0.02	-0.02	-0.03	-0.03	-0.29***	-0.27***	-0.29***	-0.27***	-0.29***	-0.27***
	(0.03)	(0.03)	(0.03)	(0.03)	(0.03)	(0.03)	(0.03)	(0.03)	(0.03)	(0.03)	(0.04)	(0.03)	(0.02)	(0.02)	(0.02)	(0.02)	(0.02)	(0.02)
(1) Hispanic	0.18***	0.18***	0.13***	0.14***	0.10**	0.11**	0.08**	0.08**	0.04	0.03	0.05	0.05	-0.09***	-0.11***	-0.05*	-0.07**	-0.08**	-0.09***
	(0.03)	(0.03)	(0.04)	(0.04)	(0.04)	(0.04)	(0.04)	(0.04)	(0.04)	(0.04)	(0.05)	(0.05)	(0.02)	(0.02)	(0.03)	(0.03)	(0.03)	(0.03)
(1) Race non-white, Black, or Hispanic	0.02	0.02	0.01	0.02	0.04	0.05	0.29***	0.28***	0.22***	0.20***	0.20***	0.18***	-0.14***	-0.16***	-0.10**	-0.11**	-0.08	-0.10**
	(0.05)	(0.05)	(0.07)	(0.06)	(0.07)	(0.07)	(0.04)	(0.04)	(0.04)	(0.04)	(0.04)	(0.04)	(0.04)	(0.04)	(0.05)	(0.04)	(0.05)	(0.05)
(1) Summer measurement	-0.06	-0.06	-0.07*	-0.07*	-0.08*	-0.08*	-0.02	-0.01	-0.02	-0.01	-0.01	-0.01	0.00	-0.00	0.00	-0.00	0.01	0.01
	(0.04)	(0.04)	(0.04)	(0.04)	(0.04)	(0.04)	(0.05)	(0.05)	(0.05)	(0.05)	(0.05)	(0.05)	(0.02)	(0.02)	(0.03)	(0.02)	(0.03)	(0.02)
ln(Real HH Income)			-0.05***	-0.05***	-0.05***	-0.04***			0.00	0.01	-0.00	-0.00			-0.00	-0.00	0.01	0.01
			(0.01)	(0.01)	(0.01)	(0.01)			(0.02)	(0.02)	(0.02)	(0.02)			(0.01)	(0.01)	(0.01)	(0.01)
(1) College Ed.			-0.07**	-0.07**	-0.03	-0.03			0.06**	0.06**	0.05*	0.05*			-0.02	-0.02	-0.01	-0.01
			(0.03)	(0.03)	(0.04)	(0.03)			(0.03)	(0.03)	(0.03)	(0.03)			(0.03)	(0.03)	(0.03)	(0.03)
Constant	0.19***	0.21***	0.81***	0.80***	0.76***	0.74***	-0.20***	-0.20***	-0.27	-0.29	-0.20	-0.20	-0.10***	-0.09***	-0.05	-0.06	-0.13	-0.16
	(0.05)	(0.06)	(0.16)	(0.17)	(0.17)	(0.17)	(0.06)	(0.06)	(0.21)	(0.20)	(0.21)	(0.21)	(0.03)	(0.03)	(0.14)	(0.13)	(0.15)	(0.15)
Observations	7,309	7,147	7,309	7,147	6,721	6,578	7,248	7,183	7,248	7,183	6,679	6,625	7,246	7,022	7,246	7,022	6,672	6,463
R-squared	0.197	0.185	0.201	0.190	0.210	0.201	0.055	0.047	0.058	0.050	0.065	0.057	0.050	0.047	0.051	0.048	0.057	0.053

Standard errors in parentheses. Models utilize weights to account for the population sampling probabilities of the NHANES, and use linearized standard errors.

Robustness of estimates to alternative samples with different levels of winsorization tested, 6% (top and bottom 3%) and 10% (top and bottom 10%).
All models conditional on age, medication use, smoking, and survey wave. SES models conditional on household size and nativity. Health models conditional on Height, waist circumference, hours of sleep, iodine levels, grams of carbohydrates per day, grams of protein per day, grams of fat per day, and %HbA1c.

Table S11. Robustness of Relationships between Free T3 and likelihood of being unemployed to winsorization

	[1]	[2]	[3]	[4]	[5]	[6]	[7]	[8]	[9]	[10]	[11]	[12]
	Base Model		Add SES		Add Health		Base Model		Add SES		Add Health	
	OLS - 6%	Logit - 6%	OLS - 6%	Logit - 6%	OLS - 6%	Logit - 6%	OLS - 10%	Logit - 10%	OLS - 10%	Logit - 10%	OLS - 10%	Logit - 10%
Free T3	0.01	1.01	0.01	1.03	0.00	0.99	0.01	0.99	0.01	1.01	-0.00	0.97
	(0.01)	(0.07)	(0.01)	(0.08)	(0.01)	(0.08)	(0.01)	(0.07)	(0.01)	(0.07)	(0.01)	(0.07)
T3 * Age	0.03***	1.20***	0.02***	1.13**	0.01**	1.08	0.03***	1.19***	0.02***	1.12**	0.01*	1.08
	(0.01)	(0.05)	(0.01)	(0.05)	(0.01)	(0.05)	(0.01)	(0.05)	(0.01)	(0.06)	(0.01)	(0.05)
Age (10 years)	-0.13***	0.46***	-0.22**	0.47	-0.18*	0.50	-0.13***	0.46***	-0.23**	0.44	-0.19*	0.47
	(0.01)	(0.04)	(0.10)	(0.33)	(0.10)	(0.39)	(0.01)	(0.04)	(0.10)	(0.32)	(0.10)	(0.38)

Models utilize weights to account for the population sampling probabilities of the NHANES, and use linearized standard errors.
Coefficients come from a fully interacted model with age and the described covariates. Standard errors in parentheses.
T3, T4, and TSH outcomes expressed as standard deviations
Robustness of estimates to alternative samples with different levels of winsorization tested, 6% (top and bottom 3%) and 10% (top and bottom 10%).
*** p<0.01, ** p<0.05, * p<0.1

Table S12. Robustness of Demographic, Socio-economic, and Health Relationships to Dropping Hypo/Hyper-thyroid adults

	[1]	[2]	[3]	[4]	[5]	[6]	[7]	[8]	[9]
Model Specification:	Base			Base & SES			Base, SES, Health and Health Behavior		
	Free T3	Free T4	TSH	Free T3	Free T4	TSH	Free T3	Free T4	TSH
(1) Male	0.51***	0.08***	0.01	0.52***	0.08***	0.01	0.56***	0.05	0.02
	(0.02)	(0.02)	(0.02)	(0.02)	(0.03)	(0.02)	(0.03)	(0.04)	(0.04)
(1) Black	-0.13***	-0.04	-0.29***	-0.17***	-0.05	-0.29***	-0.19***	-0.08	-0.29***
	(0.04)	(0.05)	(0.02)	(0.04)	(0.05)	(0.02)	(0.04)	(0.05)	(0.02)
(1) Hispanic	0.18***	0.09**	-0.10***	0.12**	0.03	-0.07**	0.06	0.05	-0.09***
	(0.04)	(0.04)	(0.02)	(0.05)	(0.05)	(0.03)	(0.06)	(0.05)	(0.03)
(1) Race non-white, Black, or Hispanic	-0.03	0.34***	-0.15***	-0.03	0.27***	-0.12***	0.01	0.24***	-0.10**
	(0.06)	(0.06)	(0.04)	(0.07)	(0.05)	(0.04)	(0.08)	(0.05)	(0.04)
(1) Summer measurement	-0.10**	-0.02	-0.00	-0.11**	-0.01	-0.00	-0.12**	-0.02	0.01
	(0.04)	(0.06)	(0.02)	(0.04)	(0.06)	(0.02)	(0.04)	(0.06)	(0.02)
ln(Real HH Income)				-0.05***	-0.02	-0.01	-0.05***	-0.02	0.01
				(0.02)	(0.02)	(0.01)	(0.02)	(0.02)	(0.01)
(1) College Ed.				-0.10***	0.08***	-0.02	-0.05	0.08**	-0.01
				(0.04)	(0.03)	(0.03)	(0.04)	(0.03)	(0.03)
Constant	0.32***	-0.13*	-0.14***	0.95***	0.08	-0.08	0.92***	0.14	-0.18
	(0.06)	(0.07)	(0.03)	(0.20)	(0.22)	(0.13)	(0.21)	(0.23)	(0.15)
Observations	7,181	7,181	7,181	7,181	7,181	7,181	6,611	6,611	6,611
R-squared	0.218	0.058	0.051	0.223	0.061	0.052	0.236	0.071	0.056

Standard errors in parentheses. Models utilize weights to account for the population sampling probabilities of the NHANES, and use linearized standard errors.
All models conditional on age, medication use, smoking, and survey wave. SES models conditional on household size and nativity. Health models conditional on Height, waist circumference, hours of sleep, iodine levels, grams of carbohydrates per day, grams of protein per day, grams of fat per day, and %HbA1c.
Dropping individuals with hyperthyroidism (TSH<0.4mIU/L) or hypothyroidism (TSH>4.1mIU/L)

Table S13. Relationships between Thyroid Hormones and Mortality, Age 50, dropping adults over clinical thresholds

	[1]	[2]	[3]	[4]	[5]	[6]
	Base Model				Add SES	Add Health
	Mortality					
Free T3	0.86***			0.84***	0.84***	0.89*
	(0.05)			(0.05)	(0.05)	(0.06)
Free T4		1.25***		1.27***	1.28***	1.27***
		(0.06)		(0.06)	(0.05)	(0.06)
TSH			1.06	1.10*	1.10*	1.09
			(0.05)	(0.06)	(0.06)	(0.06)

Models utilize weights to account for the population sampling probabilities of the NHANES, and use linearized standard errors. Hazard Ratios come from a Cox proportional hazards model conditional on the described covariates. Standard errors in parentheses.

Sample restricted to over 50 years old to focus on mortality. Mortality data through 2019.

Dropping individuals with hyperthyroidism (TSH<0.4mIU/L) or hypothyroidism (TSH>4.1mIU/L)

T3, T4, and TSH outcomes expressed as standard deviations

*** p<0.01, ** p<0.05, * p<0.1

Table S14. Alternate Linear Age Specification for Demographic, Socio-economic, and Health Relationships with Standardized Thyroid-Axis Hormones

Model Specification:	[1]	[2]	[3]	[4]	[5]	[6]	[7]	[8]	[9]	[10]	[11]	[12]
	Base			Base & SES			Base, SES, Health and Health Behavior			Other Thyroid Hormones		
	Free T3	Free T4	TSH	Free T3	Free T4	TSH	Free T3	Free T4	TSH	Free T3	Free T4	TSH
Free T3											0.16***	-0.01
											(0.02)	(0.01)
Free T4										0.14***		-0.11***
										(0.01)		(0.02)
TSH										-0.01	-0.10***	
										(0.01)	(0.01)	
Age in Years	-0.02***	0.00*	0.01***	-0.02***	0.00	0.01***	-0.02***	0.00	0.01***	-0.02***	0.01***	0.01***
	(0.00)	(0.00)	(0.00)	(0.00)	(0.00)	(0.00)	(0.00)	(0.00)	(0.00)	(0.00)	(0.00)	(0.00)
(1) Male	0.51***	0.07***	0.01	0.52***	0.07***	0.01	0.57***	0.06	0.01	0.56***	-0.03	0.03
	(0.02)	(0.03)	(0.03)	(0.02)	(0.03)	(0.03)	(0.04)	(0.04)	(0.05)	(0.04)	(0.04)	(0.05)
(1) Black	-0.12***	-0.04	-0.40***	-0.16***	-0.06	-0.41***	-0.19***	-0.08	-0.39***	-0.18***	-0.09*	-0.40***
	(0.04)	(0.04)	(0.03)	(0.04)	(0.05)	(0.04)	(0.04)	(0.05)	(0.04)	(0.04)	(0.05)	(0.04)
(1) Hispanic	0.18***	0.09**	-0.13***	0.12**	0.03	-0.10**	0.06	0.04	-0.12**	0.06	0.02	-0.11**
	(0.04)	(0.04)	(0.03)	(0.05)	(0.05)	(0.05)	(0.05)	(0.06)	(0.05)	(0.05)	(0.06)	(0.05)
(1) Race non-white, Black, or Hispanic	-0.01	0.33***	-0.16***	-0.01	0.26***	-0.13**	0.03	0.23***	-0.10	-0.00	0.21***	-0.08
	(0.06)	(0.05)	(0.04)	(0.07)	(0.05)	(0.05)	(0.08)	(0.05)	(0.06)	(0.08)	(0.05)	(0.06)
(1) Summer measurement	-0.09*	-0.03	0.00	-0.09**	-0.03	0.00	-0.11**	-0.03	0.01	-0.10**	-0.01	0.01
	(0.05)	(0.06)	(0.03)	(0.05)	(0.06)	(0.03)	(0.05)	(0.06)	(0.03)	(0.05)	(0.06)	(0.03)
ln(Real HH Income)				-0.05**	-0.04**	-0.03**	-0.04**	-0.04**	-0.01	-0.03*	-0.04*	-0.01
				(0.02)	(0.02)	(0.01)	(0.02)	(0.02)	(0.01)	(0.02)	(0.02)	(0.02)
(1) College Ed.				-0.10***	0.06*	0.01	-0.05	0.05	0.02	-0.06	0.06*	0.03
				(0.03)	(0.03)	(0.04)	(0.04)	(0.03)	(0.05)	(0.04)	(0.03)	(0.05)
Constant												
	-0.22***	-0.22***	0.21***	0.31	0.21	0.53***	0.25	0.25	0.27	0.21	0.24	0.30*
Observations	(0.05)	(0.06)	(0.04)	(0.20)	(0.21)	(0.16)	(0.21)	(0.22)	(0.17)	(0.21)	(0.21)	(0.17)
R-squared												

Standard errors in parentheses. Models utilize weights to account for the population sampling probabilities of the NHANES, and use linearized standard errors.
All models conditional on age, medication use, smoking, and survey wave. SES models conditional on household size and nativity. Health models conditional on Height, waist circumference, hours of sleep, iodine levels, grams of carbohydrates per day, grams of protein per day, grams of fat per day, and %HbA1c.
